# Phase I study of bortezomib and cetuximab in patients with solid tumours expressing epidermal growth factor receptor

**DOI:** 10.1038/sj.bjc.6605043

**Published:** 2009-04-28

**Authors:** A Z Dudek, K Lesniewski-Kmak, N J Shehadeh, O N Pandey, M Franklin, R A Kratzke, E W Greeno, P Kumar

**Affiliations:** 1 Division of Hematology, Department of Medicine, Oncology and Transplantation, Comprehensive Cancer Center, University of Minnesota, Minneapolis, MN, USA; 2 Pomorskie Centrum Onkologii, Oddzial Onkologii i Radioterapii, Gdynia, Poland

**Keywords:** EGFR, proteasome inhibition, cetuximab, bortezomib, epithelial solid tumours

## Abstract

Bortezomib inhibits nuclear factor-*κ*B (NF-*κ*B). Cetuximab is a chimeric mouse–human antibody targeted against epidermal growth factor receptor (EGFR). We hypothesised that concomitant blockade of NF-*κ*B and EGFR signalling would overcome EGFR-mediated resistance to single-agent bortezomib and induce apoptosis through two molecular pathways. The aim of this phase I trial was to establish the maximum tolerated dose (MTD) for bortezomib plus cetuximab in patients with EGFR-expressing epithelial tumours. The 21-day treatment cycle consisted of bortezomib administered on days 1 and 8 through dose escalation (1.3–2 mg m^−2^). Cetuximab was delivered at a dose of 250 mg m^−2^ on days 1, 8 and 15 (400 mg m^−2^ day 1 cycle 1). A total of 37 patients were enroled and given a total 91 cycles. No grade ⩾3 haematological toxicity was noted. Non-hematological grade ⩾3 toxicities included fatigue (22% of patients), dyspnoea (16%) and infection (11%). The MTD was not reached at the highest tested bortezomib dose (2.0 mg m^−2^). Efficacy outcomes included disease progression in 21 patients (56.7%) and stable disease (SD) at 6 weeks in 16 patients (43.3%). Five of the six patients with SD at 12 weeks were diagnosed with cancers of the lungs or head and neck. This combination therapy was moderately effective in extensively pretreated patients with non-small cell lung or head and neck cancers and warrants further investigation.

Single-agent targeted therapy has proven effective in blocking the growth and spread of many different tumour types. However, one of the main causes of its limited application in cancer treatment is that most tumours are not dependent on a single molecular pathway and are capable of circumventing single-target inhibition through the development of several mechanisms of resistance. Earlier efforts to slow the emergence of resistance have been achieved with combinations of traditional chemotherapies. Similarly, combinations of targeted agents acting on different molecular pathways involved in cancer pathogenesis may provide not only an additive effect, but may also lead to apoptotic synergy and delay the onset of resistance.

Of the vast number of possible combinations of targeted agents, preclinical data suggest that there could be a potential benefit from combining bortezomib and cetuximab (Cascone *et al*, 2008; Sloss *et al*, 2008; Wagenblast *et al*, 2008; Wagenblast *et al*, 2009). Bortezomib is the first drug in a new class of targeted therapies called proteasome inhibitors. The mechanism of action of bortezomib is not entirely known. Bortezomib inhibits the orderly degradation of cellular proteins by the ubiquitin–proteasome pathway, leading to the inactivation of the anti-apoptotic transcriptional regulator, nuclear factor-*κ*B (NF-*κ*B) (Rajkumar *et al*, 2005). Inactivation of NF-*κ*B could be one of the mechanisms by which bortezomib induces cell-cycle arrest and apoptosis in solid tumours (Mitsiades *et al*, 2006). Other mechanisms in non-hematological malignancies may include induction of apoptosis by Bim and Bik protein upregulation (Li *et al*, 2008), reduction of bcl-2 levels (Mortenson *et al*, 2005), induction of NOXA protein (Fribley *et al*, 2006) or reactive oxygen species generation (Ling *et al*, 2003). Subtoxic concentrations of bortezomib potently sensitise multiple myeloma cell lines to DNA-damaging chemotherapeutic agents, including cells resistant to these drugs (Mitsiades *et al*, 2003). Bortezomib also abolishes cell adhesion-mediated drug resistance. Consistent with these preclinical data, bortezomib has been effective in clinical trials for myeloma (Richardson *et al*, 2003, 2005; Jagannath *et al*, 2004) and responses have been documented in solid tumours, including renal cancer (Kondagunta *et al*, 2004), small cell and non-small cell lung cancer (Lara *et al*, 2006a, 2006b), and prostate cancer (Hainsworth *et al*, 2007).

Cetuximab is a chimeric mouse–human antibody targeted against epidermal growth factor receptor (EGFR). Competitive binding of cetuximab to EGFR prevents ligand-induced tyrosine kinase activation and induces receptor downregulation. In clinical trials, cetuximab is active in colorectal cancer (Rosenberg *et al*, 2002; Cunningham *et al*, 2004; Folprecht *et al*, 2004; Lenz *et al*, 2004; Rougier *et al*, 2004; Saltz *et al*, 2004) and in concomitant treatment with radiotherapy in head and neck cancer (Bonner *et al*, 2006). Upregulation of cell-surface EGFR by bortezomib was indicated in earlier studies and recently reported in squamous-cell cancer lines (Lorch *et al*, 2007). This observation strongly suggests that bortezomib could sensitise cancer cells to EGFR inhibition. However, another preclinical study has reported that the combination of a tyrosine kinase inhibitor (TKI) and bortezomib resulted in nearly complete NF-κB blockade and synergistically inhibited the proliferation of renal cancer cells, but only when TKI exposure occurred first or simultaneously with bortezomib exposure (An and Rettig, 2007). Pretreatment with bortezomib resulted in incomplete NF-κB blockade and had an antagonistic effect on proliferation. Thus, the activity of combination therapy could be schedule-dependent, and concomitant administration of both drugs could be the most optimal schedule, with the important caveat that schedule-dependent effects could vary by tumour type given the broad range of signalling events affected by bortezomib.

The combined activity of bortezomib and cetuximab in EGFR-positive solid cancers is unknown, though it is reasonable to postulate that this combination could be synergistic, given the preclinical observations that bortezomib upregulates EGFR expression in cancer cells. Although bortezomib and cetuximab have been studied extensively as single agents, the potential for combining the two agents has not been systematically explored. This Phase I dose-escalation study was designed to evaluate the safety and tolerability of combination bortezomib and cetuximab, and characterize the preliminary effectiveness of the drug combination in patients with refractory solid tumours expressing EGFR.

## Patients and methods

### Study design

This study was designed as a prospective, single-center, dose-escalation phase I study at the University of Minnesota Masonic Cancer Center. The primary objective was to determine the maximum tolerated dose (MTD) of bortezomib, when given with a fixed dose of cetuximab, in the treatment of patients with solid tumours expressing EGFR. The secondary objective was to obtain preliminary information regarding the anti-tumor activity of bortezomib and cetuximab, when given in combination. The study was reviewed and approved by University of Minnesota Institutional Review Board. Each patient provided written informed consent before participation. This trial was registered at the US National Institutes of Health (http://www.clinicaltrials.gov), unique identifier NCT00622674.

### Patient eligibility

All patients were ⩾18 years old with histologically confirmed advanced solid cancer refractory to standard treatment and at least one EGFR-positive tumour specimen determined by immunohistochemical staining. Expression of EGFR was assessed by pathologists at the University of Minnesota. An ECOG performance status of 0–1 and a life expectancy of at least 12 weeks were required. Earlier systemic chemotherapy, immunotherapy or biological therapy was allowed, except for earlier treatment with bortezomib and/or cetuximab. Patients were required to complete earlier radiation or systemic therapy at least 14 days before study entry and at least 30 days for investigational agents. Any grade >1 toxicity had to be resolved before study enrolment. Adequate organ function within 14 days of study enrolment was required, including adequate bone marrow reserve: absolute neutrophil count (ANC) ⩾1.5 × 10^9^ l^−1^, platelets >100 × 10^9^ l^−1^ and haemoglobin >9 g per 100 ml, and hepatic function: bilirubin <1.5 times the upper limit of normal ( × ULN), alkaline phosphatase (ALP), aspartate transaminase (AST) and alanine transaminase (ALT) <3.0 × ULN (ALP, AST and ALT <5 × ULN was acceptable if liver had tumour involvement). A calculated or measured creatinine clearance of >30 ml min^−1^ was required within 14 days before enrolment. Disease status had to be that of measurable or non-measurable disease, as defined by RECIST criteria.

### Treatment schedule

The treatment schedule consisted of a 21-day cycle with bortezomib given on days 1 and 8 and cetuximab given on days 1, 8 and 15 until unresolved dose-limiting toxicity (DLT), disease progression (DP) or a maximum of six cycles was completed. For all treatment cycles, bortezomib was administered before cetuximab on days 1 and 8. Day 1 treatment was administered only in patients with an ANC ⩾1500 mm^−3^ and platelets ⩾100 000 mm^−3^. Doses were recalculated before each treatment cycle on the basis of the patient's body surface area. Bortezomib was given at several escalating doses by intravenous (i.v.) push over 3–5 s followed by a standard saline flush or through a running i.v. line. Patients received cetuximab as an i.v. infusion through infusion pump or gravity drip. The infusion rate never exceeded 10 mg min^−1^. A loading dose of cetuximab (400 mg m^−2^) was infused over 90 min on day 1, cycle 1 followed by a maintenance dose of 250 mg m^−2^ infused over 60 min on days 8 and 15 of cycle 1 and days 1, 8 and 15 of subsequent cycles. Premedication with H_1_ antagonists was given before each dose of cetuximab.

Bortezomib was provided by a partial sponsor of the study, Millenium Pharmaceuticals (Cambridge, MA, USA), and released through the Investigational Pharmacy of the University of Minnesota (Minneapolis, MN, USA).

### Bortezomib dose escalation

Eight dose levels were studied with a minimum of three patients in each dose level. The starting dose of bortezomib was 1.3 mg m^−2^ with 0.1 increment increases with each successive dose level up to a maximum of 2.0 mg m^−2^. All patients within a dose level had to complete a minimum of one cycle of treatment and experience no DLT before the next group of three patients could be enroled in the next higher dose level. If one DLT occurred in cycle 1, three additional patients had to be treated at the same dose level and all complete the first cycle without DLT.

Toxicity was assessed during the first cycle. Dose escalation for subsequent cycles in the same patient (intra-patient dose escalation) was not permitted. DLT was defined as treatment-related grade ⩾4 haematological toxicity or grade ⩾3 non-hematological toxicity occurring in the first cycle of therapy. DLT was also defined as the delay of the start of cycle 2 treatment by more than 3 weeks because of incomplete haematological recovery (ANC ⩽1500 mm^3^ or platelets ⩽100 000 mm^3^) or unresolved non-hematological ⩾2 toxicity. Adverse events were classified according to NCI's Common Terminology Criteria for Adverse Events V 3.0 (CTCAE). If two or more DLTs occurred at a given dose level, the dose just below would define the MTD and would be considered the recommended dose for future phase II trials.

### Efficacy assessment and follow-up care

Restaging procedures, including imaging studies, were carried out during the 30-day period before starting therapy and after every two cycles of treatment (every 6 weeks). Study follow-up was halted after the completion of six cycles of therapy, because the primary end point was to assess safety and establish the maximally tolerated dose. Patients benefiting from therapy were given the choice of continuing beyond 6 cycles.

### Statistical methods

Comparisons between the groups with different EGFR expression, and analysis of skin rash correlation with response, were carried out using the Fisher's exact test for categorical variables.

## Results

### Patient characteristics

A total of 37 patients were enroled in this study between November 2005 and August 2008. The majority of patients were male (62.9%), had an ECOG performance status of 1 (51.4%), were diagnosed with lung cancer (40%) and had received more than two types of earlier systemic therapy (62.9%). Patient characteristics are summarised in Table 1.

### Bortezomib dose-escalation findings

At least three patients were enroled at each dose level of bortezomib. A DLT was first observed in one of three patients in cohort 4 (1.6 mg m^−2^), but was not observed in the next three patients at the same dose level. This patient developed grade 3 nausea, mucositis, vomiting, dehydration and dysphagia in the first cycle of therapy. The only other DLT was seen in cohort 8 (2 mg m^−2^), in a patient who developed respiratory failure because of pneumonia and died after the first cycle of therapy. This patient was the twelfth of the 13 patients enroled in cohort 8, the highest dose level of bortezomib. Two patients in cohort 8 also developed grade 4 urticaria on day 1 of cycle 1 and could not be re-exposed to cetuximab. These two patients were replaced because allergic and anaphylactic reactions to cetuximab are well-known and do not constitute a DLT. No MTD was reached in the study.

### Toxicity assessment

Overall, the regimen of bortezomib and cetuximab was well-tolerated. A total of 91 cycles of therapy was administered with a median of two cycles per patient (range: 1–6). Three patients completed six cycles (8.1%). Treatment delays were required in 23 patients (62%) because of low platelet count or other non-hematological symptoms, such as Gram-negative bacteremia, constipation, neuropathic pain, fatigue or muscle weakness.

All treatment-related grade ⩾3 toxicities are presented in Table 2. No subject in the trial developed grade 3 or 4 haematological toxicity. The most common grade 1 and 2 haematological side effects were decreased haemoglobin (54% of patients) and lymphopenia (22% of patients). All grade 3 or 4 toxicities were non-hematological in origin, the most common of which were fatigue (22% of patients), dyspnoea (16%), infection (11%), dehydration (11%), constipation (8%), nausea (8%) and muscle weakness (5%). Grade 3 cellulitis was observed at 1.3 mg m^−2^ in a 58-year-old male patient with pyriform sinus cancer. Renal failure occurred in two patients at doses of 1.5 and 1.9 mg m^−2^. At the higher dose, renal failure was fatal for a 52-year-old female with bladder cancer. The most commonly observed grade 1 and 2 non-hematological side effects were fatigue (81% of patients), skin rash (78%), anorexia (57%), vomiting (54%), nausea (51%), constipation (51%), diarrhoea (49%), peripheral neuropathy (35%), infection (22%) and dyspnoea (16%).

### Tumour response

Of the 37 enroled patients, only four patients (10.8%) had stable disease (SD) after six cycles. Stable disease was achieved in six patients (16.2%) after four cycles and in 16 patients (43.3%) after two cycles. Disease progression was observed in 21 patients (56.7%) after two cycles and in 28 patients (75.6%) after four cycles. Response data is not available for three patients who withdrew before completing the first cycle of therapy. Stable disease was seen at different dose levels: 1.3, 1.4, 1.5, 1.9 and 2 mg m^−2^. Although SD was seen in different cancer types, five of the six patients were diagnosed with cancers of the lungs or head and neck. One other patient experiencing SD was diagnosed with skin cancer. The degree of EGFR staining did not correlate with response (*P*=0.562) (Table 3). Although SD was seen in 50% of patients with skin rash and only 12.5% without, the presence of skin rash did not correlate significantly with SD (*P*=0.104).

## Discussion

In this first phase I study of bortezomib with cetuximab, the MTD was not achieved in the range of tested doses. An earlier phase I study established the DLT for single-agent, once weekly bortezomib at 2 mg m^−2^ (Papandreou *et al*, 2004). Therefore, we believe that combination of bortezomib and cetuximab can be used safely at the highest recommended doses for each single agent tested. None of the haematological toxicities experienced by our patient population were serious (only grade 1/2). In addition, the expected toxicities related to cetuximab were predominantly grade 1 or 2 (skin rash and diarrhoea reported in 81 and 51% of patients, respectively), and these did not worsen in frequency or grade by the addition of bortezomib.

In our study, no patient achieved complete or partial response, and only six patients experienced SD for at least 12 weeks. A total of 28 subjects had progressive disease, which is a disappointing outcome. A key finding of our study, which warrants further investigation, is that every patient who achieved a clinical benefit was diagnosed with either cancer of the lungs or head and neck. These responses were seen throughout different dose levels of bortezomib. The identification of another disease marker could possibly enrich the study population with potential responders for subsequent clinical trials of this combination of targeted agents. Recent data of lack of response of K-ras-mutated colorectal tumours to cetuximab (Karapetis *et al*, 2008) may help rationalise the use of this selection criteria in future studies with bortezomib and cetuximab. Another observation, perhaps not surprising in view of the colorectal data (Chung *et al*, 2005), is that there was no apparent correlation between patient responses and intensity of EGFR expression. Our goal in requiring EGFR expression was to exclude malignancy without an EGFR target, and thus, patients who would not benefit from cetuximab. Since the initiation of our study, it has become clear that response to EGFR-targeted therapy is not correlated with EGFR overexpression in the context of colorectal carcinoma (Chung *et al*, 2005). None of our patients in this study, however, had colorectal carcinoma. The current standard of not testing colorectal tumours for EGFR expression may not apply for other epithelial tumour histologies. It is possible that low serum ligand levels of EGF or TGF*α* would correlate better with response, as suggested by one study (Han *et al*, 2009). Recent phase II and III trials have shown that the presence of a K-ras mutation in tumours predicts response to EGFR-targeted therapy (Allegra *et al*, 2009). Intensive research, attempting to correlate the benefits from EGFR-targeted therapy with EGFR presence, EGFR mutation (NSCLC), EGFR gene amplification (NSCLC), EGFR pathway dependence or downstream mutations (conveying resistance to EGFR therapy, such as K-ras mutation in colorectal carcinoma) is currently underway, and most likely will provide different answers for different cancer histologies.

In conclusion, treatment with bortezomib and cetuximab resulted in a tolerable toxicity profile at the highest dose level of bortezomib tested. The MTD was not reached. Although our study tested concomitant administration, the optimal timing of both drugs administration remains to be determined and could be influenced by tumour type. We propose using bortezomib at a dose of 2 mg m^−2^ on days 1 and 8 combined with immediate administration of cetuximab at a 400 mg m^−2^ loading dose on day 1, followed by 250 mg m^−2^ weekly, in future phase II trials of epithelial cancers.

## Figures and Tables

**Table 1:** Patient characteristics

	***n*=37**
Median age	56 (range: 31–68)
	
*Gender*	
Male	24 (64.9%)
Female	13 (35.1%)
	
*Race*	
Caucasian	32 (86.5%)
Asian	4 (10.8%)
Black	1 (2.7%)
	
*Primary tumour site*	
Lung	16 (43.2%)
Head/neck	6 (16.2%)
Kidney	4 (10.8%)
Pancreas	3 (8.1%)
Bladder	2 (5.4%)
Skin	1 (2.7%)
Oesophagus	1 (2.7%)
Ovary	1 (2.7%)
Appendix	1 (2.7%)
Prostate	1 (2.7%)
Ureter	1 (2.7%)
	
*Lung cancer histology*	
Adenocarcinoma	11 (29.7%)
Adenosquamous cell	1 (2.7%)
Squamous cell	2 (5.4%)
Small cell	2 (5.4%)
	
*ECOG status*	
0	18 (48.6%)
1	19 (51.4%)
	
*Earlier systemic therapy*	
0	1 (2.7%)
1	8 (21.6%)
2	4 (10.8%)
3 and more	24 (64.9%)
	
*Earlier surgery*	
Yes	21 (56.8%)
No	16 (43.2%)
	
*Earlier radiotherapy*	
Yes	20 (54%)
No	17 (46%)

Abbreviation: ECOG=Eastern Cooperative Oncology Group.

**Table 2:** Grade ⩾3 toxicity for each bortezomib dose level

			**Incidence of toxicity^b^**			
**Dose (mg m^−2^)**	**No. of patients**	**No. of cycles^a^ (median, range)**	**Patients (%)**	**Cycles (%)**	**Toxicity type/grade (total no. of patients, cycles)**	
1.3	3	11 (4, 1–6)	2 (67)	6 (55)	Fatigue G3 (1, 2) Infection G3 (2, 4) Haemorrhage G4 (1, 1) Acneiform rash G3 (1, 1) Muscle weakness G3 (1, 2)	Peripheral neuropathy G3 (1, 2) Hypocalcaemia G3 (1, 1) Hypophosphataemia G3 (1, 1) Chills G3 (1, 1)
1.4	3	6 (2, 1–3)	2 (67)	2 (33)	Dyspnoea G3 (1, 1) Constipation G3 (1, 1)	
1.5	3	9 (2, 1–6)	2 (67)	2 (22)	Hyperkalaemia G3 (1, 1) BUN elevation G3 (1, 1) Mucositis G4 (1, 1) Vomiting G3 (1, 1)	Low bicarbonates G3 (1, 1) Creatinine elevation G4 (1, 1)
1.6	6	17 (4, 1–4)	3 (50)	4 (24)	Fatigue G3 (2, 2) Dyspnoea G3 (2, 2) Vomiting (1, 1) Dehydration G3 (1, 1)	Dysphagia G3 (1, )1
1.7	3	6 (2, 2–2)	2 (67)	3 (50)	Fatigue G3 (2, 2) Infection G3 (1, 1) Pain G3 (1, 2) AST elevation G3 (1, 1)	Infection G3 (1, 1)
1.8	3	6 (2, 2–2)	2 (67)	2 (33)	Muscle weakness G3 (1, 2)	
1.9	3	13 (4, 3–6)	2 (67)	3 (23)	Fatigue G3 (1, 1) Constipation G3 (1, 2) Dehydration G3 (2, 1) Abdominal pain G3 (1, 1)	Anorexia G3 (1, 1) Nausea G3 (1, 1) Death G5 (1, 1)
2.0	13	23 (2, 1–4)	9 (69)	10 (43)	Fatigue G3 (3, 3) Infection G3 (1, 1) Dyspnoea G3 (3, 4) Constipation G3/4 (1, 2) Dehydration G3 (1, 1) Nausea G3 (2, 2) Diarrhoea G3 (1, 1)	Dizziness G3 (1, 1) Peripheral neuropathy G3 (1, 1) Low blood pressure G3 (1, 1) Death G5 (1, 1)

Abbreviations: AST=aspartate transaminase; BUN=blood urea nitrogen.

a At least two visits out of five were completed per cycle.

b Incidence of grade 1/2 toxicities was 100% for all patients at all dose levels; one patient who tolerated six cycles (1.9 mg m^−2^) did not experience any toxicities during two of these cycles.

**Table 3:** EGFR staining and response

	**After 2 weeks of treatment (two cycles)**	
**EGFR-positive stain**	**PD**	**SD**
1	5	1
2	5	8
3	9	6

Abbreviations: EGFR=epidermal growth factor receptor; PD=progressive disease; SD=stable disease.
